# Supplementary material for: Iterative improvement in the automatic modular design of robot swarms
Source: PeerJ Comput Sci. 2020 Dec 7;6:e322. doi: 10.7717/peerj-cs.322 (PMC7924708; doi:10.7717/peerj-cs.322)
Supplement: Supplemental Information 3 [file peerj-cs-06-322-s003.zip › argos3/doc/api/standalone/a00592_source.html]

ARGoS: plugins/simulator/entities/wheeled\_entity.cpp Source File


- Main Page
- Related Pages
- Namespaces
- Classes
- Files

- File List
- File Members

# plugins/simulator/entities/wheeled\_entity.cpp

Go to the documentation of this file.

```
00001 
00007 #include "wheeled_entity.h"
00008 #include <argos3/core/simulator/space/space.h>
00009 
00010 namespace argos {
00011 
00012    /****************************************/
00013    /****************************************/
00014 
00015    CWheeledEntity::CWheeledEntity(CComposableEntity* pc_parent,
00016                                   size_t un_num_wheels) :
00017       CEntity(pc_parent),
00018       m_unNumWheels(un_num_wheels) {
00019       m_pcWheelPositions = new CVector3[m_unNumWheels];
00020       ::memset(m_pcWheelPositions, 0, m_unNumWheels * sizeof(CVector3));
00021       m_pfWheelRadia = new Real[m_unNumWheels];
00022       ::memset(m_pfWheelRadia, 0, m_unNumWheels * sizeof(Real));
00023       m_pfWheelVelocities = new Real[m_unNumWheels];
00024       ::memset(m_pfWheelVelocities, 0, m_unNumWheels * sizeof(Real));
00025       Disable();
00026    }
00027 
00028    /****************************************/
00029    /****************************************/
00030 
00031    CWheeledEntity::CWheeledEntity(CComposableEntity* pc_parent,
00032                                   const std::string& str_id,
00033                                   size_t un_num_wheels) :
00034       CEntity(pc_parent, str_id),
00035       m_unNumWheels(un_num_wheels) {
00036       m_pcWheelPositions = new CVector3[m_unNumWheels];
00037       ::memset(m_pcWheelPositions, 0, m_unNumWheels * sizeof(CVector3));
00038       m_pfWheelRadia = new Real[m_unNumWheels];
00039       ::memset(m_pfWheelRadia, 0, m_unNumWheels * sizeof(Real));
00040       m_pfWheelVelocities = new Real[m_unNumWheels];
00041       ::memset(m_pfWheelVelocities, 0, m_unNumWheels * sizeof(Real));
00042       Disable();
00043    }
00044    
00045    /****************************************/
00046    /****************************************/
00047 
00048    CWheeledEntity::~CWheeledEntity() {
00049       delete[] m_pcWheelPositions;
00050       delete[] m_pfWheelRadia;
00051       delete[] m_pfWheelVelocities;
00052    }
00053    
00054    /****************************************/
00055    /****************************************/
00056    
00057    void CWheeledEntity::Reset() {
00058       ::memset(m_pfWheelVelocities, 0, m_unNumWheels * sizeof(Real));
00059    }
00060    
00061    /****************************************/
00062    /****************************************/
00063    
00064    void CWheeledEntity::SetWheel(UInt32 un_index,
00065                                  const CVector3& c_position,
00066                                  Real f_radius) {
00067       if(un_index < m_unNumWheels) {
00068          m_pcWheelPositions[un_index] = c_position;
00069          m_pfWheelRadia[un_index] = f_radius;
00070       }
00071       else {
00072          THROW_ARGOSEXCEPTION("CWheeledEntity::SetWheel() : index " << un_index << " out of bounds (allowed [0:" << m_unNumWheels << "])");
00073       }
00074    }
00075 
00076    /****************************************/
00077    /****************************************/
00078 
00079    const CVector3& CWheeledEntity::GetWheelPosition(size_t un_index) const {
00080       if(un_index < m_unNumWheels) {
00081          return m_pcWheelPositions[un_index];
00082       }
00083       else {
00084          THROW_ARGOSEXCEPTION("CWheeledEntity::GetWheelPosition() : index " << un_index << " out of bounds (allowed [0:" << m_unNumWheels << "])");
00085       }
00086    }
00087    
00088    /****************************************/
00089    /****************************************/
00090    
00091    Real CWheeledEntity::GetWheelRadius(size_t un_index) const {
00092       if(un_index < m_unNumWheels) {
00093          return m_pfWheelRadia[un_index];
00094       }
00095       else {
00096          THROW_ARGOSEXCEPTION("CWheeledEntity::GetWheelRadius() : index " << un_index << " out of bounds (allowed [0:" << m_unNumWheels << "])");
00097       }
00098    }
00099    
00100    /****************************************/
00101    /****************************************/
00102    
00103    Real CWheeledEntity::GetWheelVelocity(size_t un_index) const {
00104       if(un_index < m_unNumWheels) {
00105          return m_pfWheelVelocities[un_index];
00106       }
00107       else {
00108          THROW_ARGOSEXCEPTION("CWheeledEntity::GetWheelVelocity() : index " << un_index << " out of bounds (allowed [0:" << m_unNumWheels << "])");
00109       }
00110    }
00111    
00112    /****************************************/
00113    /****************************************/
00114    
00115    void CWheeledEntity::SetVelocities(Real* pf_velocities) {
00116       ::memcpy(m_pfWheelVelocities, pf_velocities, m_unNumWheels * sizeof(Real));
00117    }
00118 
00119    /****************************************/
00120    /****************************************/
00121 
00122    REGISTER_STANDARD_SPACE_OPERATIONS_ON_ENTITY(CWheeledEntity);
00123 
00124    /****************************************/
00125    /****************************************/
00126 
00127 }
```

---

Generated on 10 Jul 2018 for ARGoS by 
 1.6.1
